# Philosophical Transactions of the Royal Society of London, for the Year 1835

**Published:** 1836-07

**Authors:** 


ART. XIII.—*Philosophical Transactions of the Royal Society of London, for the year* 1835. Parts I. and II. 4to. pp. 365.

*Proceedings of the Royal Society.* Nos. 18-23. *From November* 20, 1834, *to February,* 1836.—8vo. pp. 73.

OWING to their very limited circulation and expensive character, the Transactions of many of the Societies in this country dedicated to the cultivation of science are very little known to the generality of the members of our profession; and, consequently, many important enquiries and discoveries relating to medical science often remain for a long time in these learned repositories, hidden from the very class of readers whom their authors are most desirous they should reach. With the view of remedying these evils, as far as in our power, we propose henceforth giving, from time to time, brief notices of all the more important papers relating to medicine, or any of its numerous branches, which may appear in the published labours of our learned Institutions.

In the present number we shall confine our attention to the most eminent of all our scientific bodies, the Royal Society; and it will at once appear, from the number and variety of the papers we shall have to notice, that natural science generally, and medical science in particular, engages no small share of the attention of that learned body.

Every one is acquainted with the publication, the first of the two which stand at the head of this Notice, it having continued to appear at pretty regular intervals, now exactly one century; and few are ignorant of the immense benefits which it has conferred on every department of science. As its name implies, it contains, at full length, the various scientific memoirs which the Council of the Society select, out of those presented to it, for publication, under the exclusive designation of the *Transactions of the Society*. The smaller publication is of recent origin and is probably unknown to many of our readers. It is a small fugitive publication, got up chiefly for the information of the members, but authenticated by the direct superintendence of the officers of the society. It comes out in small fasciculi, at irregular intervals, and gives an accurate but brief account of all the Proceedings of the Society, and an abstract, without comment, of all the papers read before it, whether destined for publication in the Transactions or not. As these abstracts are perfectly authentic, and generally of a size suited to our purpose, we propose to avail ourselves of the facilities afforded by them, in laying an outline of the memoirs before our readers; and we shall, in general, transcribe the very words of the original, without any comment of our own. We think it best, also, to make use of these abstracts in noticing the papers published at length in the Transactions, reserving to ourselves the power of examining in greater detail, and critically, at some other time, such of them as may seem to demand a more extended notice.

At the last Anniversary of the Royal Society, November 30, 1835, the number of members was 793, viz. 10 royal personages, 48 foreign members, and 735 home members: of this last class, gentlemen of the medical profession constitute a considerable proportion.

1, *On the Circulation of the Blood in Insects.* By John Tyrrell, Esq. A.M. Read January 15, 1835.

"The observations on the circulation of the blood in insects, which is a discovery of comparatively recent date, have been made almost exclusively on insects in the larva state; but the author of the present paper details a variety of observations of the same fact in insects which had arrived at their last or perfect stage of development. Among the *Myriapoda*, the circulation was traced in the *Geophilus*, and still more distinctly in the *Lithobius forficatus*. The author also detected the circulation, by the motion of globules, through the nervures of the wings of various perfect insects, namely, of some species of the *Hemerobius*, *Panorpa*, *Phryganea*, and *Ephemera*; and particularly in the *Musca domestica*, or common house-fly. The paper is accompanied by drawings of the appearances described." (P. 317.)

2, *An Experimental Inquiry into the Cause of the grave and acute Tones of the Human Voice.* By John Bishop, Esq. Read Feb. 26, 1835.

"The author considers all the theories hitherto proposed respecting the functions of the organs of the human voice, as not only unsatisfactory, but as being founded on erroneous views. He shows that the modulation of the tones of the voice is not the result of variations either exclusively in the length or in the tension of the vocal chords, or in the size of the aperture of the glottis, or in the velocity or the temperature imparted to the air in its transit through these passages. He regards the organs of the voice as combining the properties of wind and of stringed musical instruments; and shows, first, that for the production of any musical tone it is necessary that the vocal chords should previously be made mutually to approximate; and, secondly, that the muscular forces acting on the arytenoid cartilages and vocal chords are adequate not only to resist the pressure of the column of air issuing from the lungs, but also to render either the whole or certain portions of the vocal chords susceptible of vibration when traversed by the current of respired air. In proportion as these parts of the vocal chords, thus rendered vibratory, increase in length, the number of their vibrations, performed in a given time, diminishes, and the tone of the sound emitted becomes, in consequence, more grave; and, conversely, the tone is more acute as the vibrating portions of the chord are shorter: these phænomena being precisely analogous to those which take place in stringed musical instruments.

"The author concludes his paper with some observations on the comparative physiology of the voice; and on the extensive range and superior excellence of this faculty in man." (P. 323.)

3, *Discovery of the Metamorphoses in the second Type of the Cirripedes, viz. the Lepades, completing the Natural History of these singular Animals, and confirming their affinity with the Crustacea.* By J. V. Thompson, Esq. F.L.S. Read March 5, 1835.

"The discoveries made by the author of the remarkable metamorphoses which the animals composing the first family of the Cirripedes, or *Balani*, undergo in the progress of their development, and which he has published in the third number of his Zoological Researches (p. 76), are in the present paper, which is intended as a Prize Essay for one of the Royal Medals, followed up by the report of his discovery of similar changes exhibited by three species of two other genera of the second tribe of this family, namely, the *Lepades*. The larvæ of this tribe, like those of the Balani, have the external appearance of bivalve Monoculi, furnished with locomotive organs, in the form of three pairs of members, the most anterior of which are simple and the other bifid. The back of the animal is covered by an ample shield, terminating anteriorly in two extended horns, and posteriorly in a single elongated spinous process. Thus they possess considerable powers of locomotion, which, with the assistance of an organ of vision, enable them to seek their future permanent place of residence. The author is led from his researches to the conclusion that the Cirripedes do not constitute, as modern naturalists have considered them, a distinct class of animals, but that they occupy a place intermediate between the Crustacea decapoda, with which the Balani have a marked affinity, and the Crustacea entomostraca, to which the Lepades are allied; and that they have no natural affinity with the Testaceous Mollusca, as was supposed by Linnæus, and all the older systematic writers on Zoology." (P. 325.)

4, *On the supposed Existence of Metamorphoses in the Crustacea.* By J. O. Westwood, Esq., F.L.S. Read June 18, 1835.

"The author refers the principal modifications of form which occur during the progressive development of animals to the three following heads: 1st, that of an animal produced from the egg in the form which it is destined to retain through life; its only change consisting in a series of moultings of the outer envelope, attended merely by an increase of size, and not by the acquisition of new organs; 2dly, when the animal, at its exclusion from the egg, exhibits the form which it continues to possess, subject to a series of moultings, during several of the last of which certain new organs are gradually developed; and, 3dly, when the form of the animal, at its exclusion from the egg, is totally different from that under which it appears at the later periods of its existence; such change of form taking place during two or three of its general moultings, and consisting, not only in the variation of the form of the body, but also in a complete change in the nutritive and digestive systems, and in the acquisition of various new organs. This last phenomenon peculiarly characterizes what is termed a *metamorphosis*.

"It is the received opinion among naturalists that the Crustacea do not undergo metamorphoses, properly so called, and that the transformations they exhibit consist merely in the periodical shedding of the outer envelope. The object of the present paper is to establish the correctness of this opinion, in opposition to that of Mr. J. V. Thompson, who has laid claim to the discovery that the greater number of the animals belonging to the class Crustacea actually undergo metamorphoses of a peculiar kind, and of a different character from those of insects. Mr. Thompson's views are founded upon some circumstances which he has observed in certain animals of the genus *Zoea* of Bosc, and which have been recorded by Professor Slabber, and which have led Mr. Thompson to believe that, of these animals, some were the young of the *Cancer Pagurus*, or common crab, and others the young of the *Astacus Pagurus*, or common lobster; and these views are supposed by him to be corroborated by the annual peregrinations of the land crabs to the sea-side; for the purpose of depositing their eggs, rendered necessary by the aquatic habits and conformation of the young. The author proceeds to examine at length the arguments on which Mr. Thompson has founded these opinions, and adduces his reasons for concluding that they are erroneous, and that no exception occurs to the general law of development in the Crustacea, namely, that they undergo no change of form sufficiently marked to warrant the application to them of the term *metamorphosis*." (P. 341.)

5, *Continuation of the Paper on the relations between the Nerves of Motion and of Sensation, and the Brain; and more particularly on the structure of the Medulla Oblongata and of the Spinal Marrow*. By Sir C. Bell, F.R.S. Read April 30, 1835.

"The author enters into a minute anatomical investigation of the structure of the spinal cord, and of its relations with the encephalon, and with the origins of the nerves. He finds that the spinal cord is constituted, in its whole length, by six pairs of columns, namely, two posterior, two lateral, and two anterior; each column being composed of concentric layers, and invested with an external coating of cineritious substance, and all the columns being divided from each other by deep sulci, which penetrate nearly to the centre of the cord. On tracing the posterior columns in their ascent towards the encephalon, they are seen to diverge laterally at the *calamus scriptorius*, or bottom of the fourth ventricle, and to proceed into the substance of the cerebellum. Each of these posterior columns is here found to consist of two portions, the outermost being the largest; and they now constitute the *processus cerebelli ad medullam oblongatam*. This subdivision of the posterior columns may be traced throughout the whole length of the spinal cord. The lateral columns give origin to the posterior roots of the spinal nerves, and are therefore the parts subservient to sensation. In ascending towards the brain, each of these columns has a double termination; first, in the root of the fifth pair of cephalic nerves; and, secondly, in the place where both columns unite into one round cord, and mutually decussate.

"Between the lateral and the anterior columns there is interposed a layer of cineritious matter, constituting a continuous stratum from the *cauda equina* to the roots of the auditory nerves. There is also a septum, dividing the right and left tracts subservient to sensation in the region of the fourth ventricle, and apparently terminating at the point of decussation of these tracts; but, in reality, separating to allow of this decussation, and joining the central portion of the cord, which connects the posterior with the anterior columns, and extends from the *pons Varolii* to the *cauda equina*.

"The anterior columns, constituting, at their upper part, the *corpora pyramidalia*, after their union and decussation, compose the motor columns of the spinal cord. They do not, in their course, unite or decussate with the lateral or sensitive columns; decussation taking place only among the columns performing similar functions; that is, the motor columns with the motor, and the sensitive with the sensitive." (P. 331.)

6, *Observations on the Theory of Respiration.* By WILLIAM STEVENS, M.D. D.C.L. Read May 21, 1835.

"From the fact that no carbonic acid gas is given out by venous blood when that fluid is subjected to the action of the air-pump, former experimentalists had inferred that this blood contains no carbonic acid. The author of the present paper contends that this is an erroneous inference; first, by showing that serum, which had been made to absorb a considerable quantity of this gas, does not yield it upon the removal of the atmospheric pressure; and next, by adducing several experiments in proof of the strong attraction exerted on carbonic acid both by hydrogen and by oxygen gases, which were found to absorb it readily through the medium of moistened membrane. By means of a peculiar apparatus, consisting of a double-necked bottle, to which a set of bent tubes were adapted, he ascertained that venous blood, agitated with pure hydrogen gas, and allowed to remain for an hour in contact with it, imparts to that gas a considerable quantity of carbonic acid. The same result had, indeed, been obtained, in a former experiment, by the simple application of heat to venous blood confined under hydrogen gas; but on account of the possible chemical agency of heat, the inference drawn from that experiment is less conclusive than from experiments in which the air-pump alone is employed. The author found that, in like manner, atmospheric air, by remaining, for a sufficient time, in contact with venous blood, on the application of the air-pump, acquires carbonic acid. The hypothesis that the carbon of the blood attracts the oxygen of the air into the fluid, and there combines with it, and that the carbonic acid thus formed is afterwards exhaled, appears to be inconsistent with the fact that all acids, and carbonic acid more especially, impart to the blood a black colour; whereas the immediate effect of exposing venous blood to atmospheric air, or to oxygen gas, is a change of colour from a dark to a bright scarlet, implying its conversion from the venous to the arterial character: hence the author infers that the acid is not formed during the experiment in question, but already exists in the venous blood, and is extracted from it by the atmospheric air. Similar experiments made with oxygen gas, in place of atmospheric air, were attended with the like results, but in a more striking degree; and tend therefore to corroborate the views entertained by the author of the theory of respiration. According to these views, it is neither in the lungs, nor generally in the course of the circulation, but only during its passage through the capillary system of vessels, that the blood undergoes the change from arterial to venous; a change consisting in the formation of carbonic acid, by the addition of particles of carbon derived from the solid textures of the body, and which had combined with the oxygen supplied by the arterial blood: and it is by this combination that heat is evolved, as well as a dark colour imparted to the blood. The author ascribes, however, the bright red colour of arterial blood, not to the action of oxygen, which is of itself completely inert as a colouring agent, but to that of the saline ingredients naturally contained in healthy blood. On arriving at the lungs, the first change induced on the blood is effected by the oxygen of the atmospheric air, and consists in the removal of the carbonic acid, which had been the source of the dark colour of the venous blood; and the second consists in the attraction by the blood of a portion of oxygen, which it absorbs from the air, and which takes the place of the carbonic acid. The peculiar texture of the lungs, and the elevation of temperature in warm-blooded animals, concur in promoting the rapid production of these changes." (P. 334.)

7, *On the Influence of the Tricuspid Valve of the Heart on the Circulation of the Blood.*" By T. W. KING, Esq., M.R.C.S. Read June 4, 1835.

"The purport of this paper is to prove experimentally that the tricuspid valve of the human heart does not, in the ordinary state of the circulation, completely prevent the reflux of blood from the ventricle into the auricle on the right side, and that the amount of regurgitation is continually varying according to the different degrees of distention of the ventricle. The author points out the anatomical differences between the auriculo-ventricular valves on the right and left sides of the heart; from the consideration of which it might have been inferred, independently of direct experiment, that while the structure of the mitral valve is adapted to close accurately all communication of the left auricle and ventricle during the contraction of the latter, that of the tricuspid valve is designedly calculated to allow, when closed, of the flow of a certain quantity of blood from the right ventricle back again into the auricle. The comparatively imperfect valvular function of these latter membranes is shown by various experiments on recent hearts, in which it was found that fluids injected through the aorta into the left ventricle were perfectly retained in that cavity, by the closing of the mitral valve; but that when the right ventricle was similarly injected through the pulmonary artery, the tricuspid valves generally allowed the escape of the fluid in streams, more or less copious, in consequence of the incomplete apposition of their margins. On repeating these experiments on different animals the author obtained similar results; but found that the imperfection of the valvular function was greater the sooner the heart was examined after the death of the animal; and that if the trials were made after the lapse of a certain time, the rigidity which gradually supervened on the muscular fibres of the heart, and of the *carneæ columnæ* attached to the margins of the valves, brought them into more complete apposition and led to the accurate closing of the passage. This effect, however, was never so perfectly accomplished in the tricuspid, as in the mitral valves.

" The author regards this peculiarity of structure in the tricuspid valve as an express provision against the mischiefs that might result from an excessive afflux of blood to the lungs, analogous to a safety-valve; and as more especially advantageous in incipient diseased enlargements of the right ventricle. He adverts to the conditions of the heart during the fœtal state of existence, in which the same necessity of guarding against excessive pressure does not occur, and where the structures are found to correspond to the variation of functions. A similar adjustment of the right auriculo-ventricular valve to the peculiar circumstances and habits of animals may also be traced by extending the inquiry to various classes of animals." (P. 337.)

8, *On the Ova of Women and Mammiferous Animals, as they exist in the Ovaries before Impregnation.* By THOMAS WHARTON JONES, Esq. Read June 18, 1835.

[We gave an account of this paper in our last Number, p. 160.

9, *On the Temperature of some Fishes of the Genus Thynnus.* By JOHN DAVY, M.D., F.R.S., Assistant Inspector of Army Hospitals. Read March 26, 1835.

" The author had occasion to observe, many years ago, that the Bonito (*Thynnus pelamys*, Cuv.) had a temperature of 99° of Fahr. when the surrounding medium was 80° 5, and that it, therefore, constituted an exception to the generally received rule that fishes are universally cold-blooded. Having found that the gills of the common Thunny of the Mediterranean (*Thynnus vulgaris*, Cuv.) were supplied with nerves of unusual magnitude, that the heart of this latter fish was very powerful, and that its muscles were of a dark red colour, he was led to conjecture that it might, like the Bonito, be also warm-blooded; and this opinion is corroborated by the testimony of several intelligent fishermen. The author endeavours to extend this analogy to other species of the same family, which, according to the reports of the fishermen of whom he made enquiries, have a high temperature, and in whose internal structure he noticed similar peculiarities as in the Thunny; namely, very large branchial nerves, furnished with ganglia of considerable size. In this respect he considers that in these fishes the branchial system of organs makes an approximation to the respiratory apparatus of the mammalia, and that it probably contributes to the elevation of temperature, resulting from the more energetic respiration which he supposes to be exercised by these organs. He, however, thinks it not improbable that these fish may possess means of generating heat peculiar to themselves, and of which at present we have no adequate idea. He conceives that the situation of the kidneys, of which a considerable portion is even higher than the stomach, and posterior to the gills, and which are of large size, and well supplied with nerves and blood-vessels, may possibly act a part in the production of an elevated temperature; but, on the whole, he is disposed to ascribe the greatest share of this effect to the superior magnitude of the branchial nerves." (P. 327.)

10, *On the Influence of the Respiratory Organs in regulating the Quantity of Blood within the Heart.* By James Wardrop, Esq. F.R.S. Read June 18, 1835.

"The author observes that the act of inspiration tends not only to favour the passage of the blood into the venæ cavæ, but also to detain it in the pulmonary vessels,—in consequence of the expansion of the lungs allowing of its more ready ingress into the pulmonary arteries, and impeding its exit by the veins—and thus retards its return to the heart. On the other hand, the collapse both of the lungs and of the parietes of the chest, during expiration, assists the transmission of arterial blood from the lungs into the left cavities of the heart, and promotes its passage into the aorta. Thus he considers inspiration as an auxiliary to the venous, and expiration to the arterial circulation; the first acting like a sucking, and the latter like a forcing pump, in aiding the power of the heart. On this principle he explains the influence exerted on the circulation, and on the action of the heart, by various modes of respiration, whether voluntary or involuntary, in different circumstances. Laughter, crying, weeping, sobbing and sighing, &c., he considers as efforts made with a view to effect certain alterations in the quantity of blood in the lungs and heart, when the circulation has been disturbed by mental emotions." (P. 342.)

11, *On the Anatomical and Optical Structure of the Crystalline Lenses of Animals.* By Sir David Brewster, K.H., LL.D., F.R.S. Read January 14, 1836.

[We can find room for only a portion of the abstract given of this curious paper, which cannot be well understood without the plates.]

"The author has examined the structure of the crystalline lens of the eye of a great variety of animals belonging to each of the four classes of vertebrata; and has communicated in this paper a detailed account of his observations, arranged according as they relate to structures more and more complex. In a former paper, published in the Philosophical Transactions for 1833, the lens of the Cod fish was taken as the type of the simplest of these structures, in as much as all the fibres of which it is composed converge, like the meridians of a globe, to two opposite points, or poles, of a spheroid or lenticular solid; both of which poles are situated in the axis of vision. The structure which ranks next in respect of simplicity is that exhibited in the salmon, among fishes; in the gecko, among reptiles; and in the hare, among mammalia. It presents at each pole two septa placed in one continuous line, in different points of which all the fibres proceeding from the one surface to the other have their origin and termination. A structure somewhat more complex is met with in the lenses of most of the mammalia, and is particularly exemplified in the lion, the tiger, the horse, and the ox. Three septa occur at each pole in the form of diverging lines inclined to one another at angles of 120°. The next degree of complexity is presented in the lens of the whale, the seal, and the bear, which contain, instead of three, four septa on each side, placed at right angles to each other in the form of a cross. In some specimens of lenses of whales and seals the author observed two septa from each pole, forming one continuous line, from each of the extremities of which proceeded two others, which were at right angles relatively to one another: so that there were in all five on each surface. The most complex structure is that of the lens of the elephant, which exhibits three primary septa diverging at equal angles from the pole, and at their extremities bifurcating into two additional septa, which are inclined to each other at angles of 60°, these latter being the real septa, to which the fibrous radiations are principally related. In some lenses of the elephant the author found the three septa immediately proceeding from the poles exceedingly short, and approaching to evanescence; so that he has no doubt that occasionally they may be found to have disappeared, and that the other six septa will then all diverge from the poles, like the radii of a hexagon, at angles of 60°." (P. 366.)

